# Leveraging GAAIN for Collaborative Dementia Research

**DOI:** 10.1002/alz70860_099974

**Published:** 2025-12-23

**Authors:** Daniel A. Nation, Trevor Lohman

**Affiliations:** ^1^ Leonard Davis School of Gerontology, University of Southern California, Los Angeles, CA, USA; ^2^ Keck School of Medicine, University of Southern California, Los Angeles, CA, USA

## Abstract

Introduction. Existing data from longitudinal cohort studies represent a critical resource that can be leveraged to gain fresh insights into Alzheimer's disease and related disorders (ADRD), including scientific premise for the repurposing of existing medications in the prevention and treatment of ADRD. One challenge is the identification and collation of cohort data appropriate to the research question.

Methods. The Global Alzheimer's Association Interactive Network (GAAIN) is the world's first federated Alzheimer's disease data sharing platform and offers researchers a rich resource for exploring data collected across different countries and modalities, including biomarker, clinical, and neuroimaging data. The GAAIN Interrogator data discovery tool allows researchers to remotely aggregate multiple datasets and conduct exploratory analyses, while preserving data ownership of the study leaders.

Results. We will present a longitudinal cohort study that demonstrates data discovery, interrogation, and analysis utilizing the GAAIN platform and other related data resources. We will showcase this example of data identification and analysis that leverages multiple longitudinal cohort studies. Specifically, we will present this example of how GAAIN data resources and analytic tools are used to identify appropriate and adequately powered samples to test longitudinal hypotheses relevant to preventative efforts.

Discussion. We show an example of the end‐to‐end process of data discovery, interrogation, and analysis facilitated by GAAIN and related data resources. This presentation is designed to inspire similar workflows that can be incorporated by ADRD investigators, increasing utilization of the data and tools shared by GAAIN to accelerate ADRD research.

